# A Narrative Review of Combat Sports Injuries With a Particular Focus on Cervical Spine Injuries

**DOI:** 10.7759/cureus.74980

**Published:** 2024-12-02

**Authors:** Dimitrios Bakirtzis, Zoi Gkiafi, Spyridon Sioutis, Ioannis Panagiotis Tolis, Alexandros Zikopoulos, Panagis M Lykoudis, Vasileios A Kontogeorgakos, Andreas Mavrogenis, Dimitrios Koulalis

**Affiliations:** 1 Orthopedic Surgery, National and Kapodistrian University of Athens, Attikon Hospital, Athens, GRC; 2 Medicine, National and Kapodistrian University of Athens, Laiko General Hospital, Athens, GRC; 3 Surgery, Attikon University Hospital, Athens, GRC

**Keywords:** cervical spine, combat sports, neck injuries, sports injuries, sports medicine

## Abstract

Combat sports encompass a wide range of disciplines, each associated with distinct injury patterns and mechanisms. From karate to wrestling, athletes face varying degrees of injury risks, with common clinical presentations including head injuries, strains, sprains, fractures, and concussions. These injuries often result from dynamic movements, physical contact, and high-impact collisions inherent to combat sports. Diagnostic approaches such as plain radiographs, computed tomography (CT), and magnetic resonance imaging (MRI) play vital roles in assessing injuries, guiding treatment decisions, and ensuring optimal outcomes for athletes. Despite advancements in imaging technology, challenges such as false-positive results underscore the importance of clinical re-evaluation to avoid unnecessary interventions and minimize the risk of long-term complications. Following injury diagnosis, the focus shifts to treatment, recovery, and prevention strategies aimed at enhancing athlete well-being and mitigating injury risks. Prompt and appropriate emergency care, including stabilization techniques and wound management, lays the groundwork for subsequent interventions. Recovery and rehabilitation processes are tailored to the specific needs of each athlete, addressing factors such as injury severity, demographics, and sport-specific demands. Quality of life considerations underscore the profound impact of severe sports injuries on physical health and psychological well-being, emphasizing the importance of holistic support and resources for athletes. Preventive measures, including rule modifications, equipment design enhancements, and concussion prevention initiatives, are vital for creating safer sporting environments and minimizing the incidence and severity of injuries. By addressing the complexities of injury management, recovery, and prevention in combat sports, stakeholders can promote athlete safety, well-being, and long-term participation in sports.

## Introduction and background

Numerous sports entail the risk of neurologic injury due to incidental head contact, but combat sports uniquely permit deliberate head contact, elevating the potential for both acute and chronic neurological damage. Studies have reported a prevalence of 17% for chronic traumatic encephalopathy (CTE) among retired boxers in the UK, illustrating the significant neurological risks associated with combat sports [1]. Acute injury rates are similar across these sports, warranting a collective consideration of their injuries. Incidence of injuries spans a wide spectrum, with reports indicating that between one-fifth to one-half of all fights in boxing, karate, and taekwondo result in injuries. Notably, boxing is predominantly associated with injuries of the upper limbs as the primary site, followed closely by the head and neck area [2,3].

## Review

Incidence and mechanisms of injuries per sport

In youth karate, head injuries are notably prevalent, with adolescent and female athletes facing higher risks compared to younger and male counterparts [4]. While junior competitions exhibit lower injury rates than elite adult events, they still surpass those of younger elite athletes with an incidence of 36.4 and 47.5 injuries per 1000 athlete-exposures (AEs), respectively, as reported in the study [5]. Notably, time-loss injuries are infrequent within this demographic group. The introduction of new competition categories in under-21 (U21) karate has yielded promising results, significantly diminishing injury rates, with the incidence ranging from 50.7 to 73.7 per 1000 AEs. This adjustment underscores a proactive approach to mitigating risks and fostering safer participation in youth karate, thus enhancing the overall well-being and longevity of athletes in the sport.

Taekwondo is associated with significant injury risks, particularly during competitive events. The incidence rates of injuries among taekwondo athletes have been consistently significant, ranking second only to football players, in the cohort of the 2008 Beijing Olympic Games, and reaching the highest in the cohort of the 2012 London Olympic Games. While there was a slight decrease during the 2016 Rio Olympic Games, the rates remained among the top in comparison to other Olympic sports. Elite athletes face a range of injury rates, from 20.6 to 139.5 per 1000 AEs, with higher rates observed during competition. Notably, while time-loss injuries are rare, the prevalence of injuries is substantial, indicating a need for comprehensive injury prevention strategies. Injury mechanisms primarily involve contact with another player (50.89%), with non-contact and overuse injuries contributing to 19.05% and 14.88% of injuries, respectively. The most common activity leading to injury is kicking (63.73%), followed by blocking (14.02%) and stepping (6.51%). Contusions are the predominant injury type, accounting for 27.38% of cases, followed by ligament sprains and strains. Despite the high incidence of injuries, most are of minimal severity [6]. Further analysis of injury data reveals that approximately 61% of injuries occur during defensive actions, indicating vulnerability during defensive maneuvers [7]. Rates of head and neck injuries in males stand at 13.3 per 1000 AE, while females experience a slightly higher rate at 14.2 per 1000 AE. Torso injuries are less frequent, comprising 4.2% and 3.1% of injuries in males and females, respectively. Similarly, upper extremity injuries are reported at 9.4% and 7.3% for males and females, respectively. However, lower extremity injuries are predominant, with rates of 21.7% and 26.6% for males and females, respectively [8]. In terms of injury types, males encounter a rate of 37.5 per 1000 AE for abrasions, contusions, and lacerations, compared to 27.9 per 1000 AE for females. Sprains and strains occur at a rate of 10.3 per 1000 AE for males and 8.7 per 1000 AE for females, while fractures are less common, with rates of 5.9 per 1000 AE for males and 3.8 per 1000 AE for females. Concussions, although less frequent, remain a concern, with rates of 13.3 per 1000 AE for males and 11.4 per 1000 AE for females [8].

Among combat sports, boxing-related injuries are more prevalent, with a significant focus on head and neck regions [3]. Studies have consistently highlighted the severe repercussions of repeated blunt-force trauma endured by boxers, leading to a spectrum of acute, subacute, and chronic neurological complications. Notably, trauma to the cranium has been linked to neurological and neuropsychological sequelae, including chronic traumatic brain injury (CTBI) [1,9]. Research indicates that up to 20% of professional boxers develop CTBI during their careers, while a staggering 40% of retired professionals exhibit symptoms of chronic brain injury. Muhammad Ali's battle with Parkinson's disease exemplifies boxing's association with neurological injuries, with a documented history of over 300 deaths in professional boxing between 1950 and 2007 [10]. The decision by the International Boxing Association (AIBA) in 2013 to disallow head guards for male senior boxers sparked controversy due to heightened awareness surrounding head injuries and concussions in sports. Traumatic brain injury (TBI) in boxing typically manifests as a result of the acceleration-deceleration mechanism, with most TBIs categorized as concussions. While concussions often result in short-term reactions from the brain, CTE presents as a progressive neurodegenerative disease, contributing to the physical shrinking of the brain and subsequent neurological disorders such as Parkinson's disease and dementia [11]. Knockouts (KOs) are recognized as the most common cause of acute neurological injuries in boxing, resulting in approximately 10 deaths per year. The sudden loss of consciousness following a KO punch often leads to temporary impairment of cerebellar and brain stem function, causing post-concussion symptoms such as memory loss, dizziness, and headaches. While many boxers appear to recover symptomatically, those with prolonged careers face heightened risks of chronic issues, including the progression of CTE. The latter, often referred to as "being punch drunk" or "dementia pugilistica," represents the most severe health concern in modern boxing. Characterized by a myriad of neuropsychiatric symptoms and motor impairments, CTE typically emerges at the end of a boxer's career or shortly after retirement. Despite its slow progression, CTE results in irreversible neuropathological changes, including pigmented cell loss and cerebral atrophy, highlighting the urgent need for enhanced preventive measures and comprehensive medical management within the sport [10].

Kickboxing, renowned for its dynamic and aggressive nature, presents a unique blend of athleticism and combat strategy. From 3481 fight participations, a total of 382 injuries were recorded, translating to an injury rate of 109.7 injuries per 1000 fight participations. Notably, kickboxing's distinctive emphasis on kicking and targeting the head as a prime target significantly influenced injury patterns, with the head/neck/face region accounting for 52.5% of all injuries, followed by the lower extremities at 39.8%. Specifically, injuries to the lower leg (23.3%), face (19.4%), and intracranial injuries (17.2%) were most common. Additionally, over 64% of injuries were superficial bruising or lacerations, indicating the need for comprehensive research into injury mechanisms and prevention strategies tailored to different kickboxing styles. Prospective studies, adjusted for exposure, are vital for ongoing monitoring and understanding of injury rates in this dynamic combat sport [12].

In wrestling, the prevalence and nature of injuries vary across different age groups. Notably, concussions emerge as the most frequent injury overall, with the head being the primary area affected within the cranio-maxillo-facial region. Of particular concern is the mechanism of injury during take-downs, which significantly increases the likelihood of hospital admissions. Over a span of 20 years analyzed in a recent study, there has been a significant increase in the average number of wrestling injuries, underscoring the need for further investigation into preventive measures [13]. A detailed examination of spine injuries in men's wrestling, spanning from the academic years 2009 to 2010 to 2013 to 2014, reveals concerning trends. A total of 57 spine injuries were identified in the NCAA-ISP database, equating to an estimated 2040 spine injuries over this timeframe. These injuries occurred at a rate of 0.71 injuries per 1000 AEs, drawing attention to the significant risk wrestlers face. Among the most commonly reported spine injuries were stingers, paralumbar muscle strains, and cervical spine disc injuries, highlighting the diverse spectrum of spinal trauma wrestlers encounter. Injuries in wrestling exhibit distinct patterns concerning timing and severity, with competitions posing a heightened risk compared to practice sessions. The postseason stands out with the highest injury rate at 1.12 per 1000 AEs, followed by the preseason and regular season. However, when compared with the regular season, the relative risk for injuries during the preseason and postseason did not show significant differences. Weight classes also play a role in injury distribution, with the 165-lb and 197-lb weight classes recording the highest injury totals. Despite the overall injury rate being relatively low, efforts to enhance injury prevention and management strategies remain imperative [14].

A study by the IDF Institutional Review Board et al. investigated traumatic injuries incurred during Krav Maga (KM) training among soldiers in the Israeli Defense Forces (IDF) over a one-year period [15]. KM, a close-range fighting system developed by the Jewish defense forces, integrates various striking and grappling techniques from martial arts such as boxing, judo, and taekwondo. With the objective of equipping soldiers with effective self-defense skills for real-world combat scenarios, KM training has become an integral component of IDF's combat readiness program. Despite its effectiveness, KM training poses inherent risks of traumatic injuries, prompting a comprehensive examination of injury patterns and severity to inform preventive strategies and enhance soldier safety during training sessions. Upper limb injuries were the most prevalent, with fingers, hands, and wrists accounting for 31% of injuries, followed by the shoulder region (16%). Lower extremity injuries were also notable, comprising 26% of reported injuries, with knees being the most commonly affected area, followed by the foot and ankle. Head, face, and neck injuries, as well as back and chest injuries, occurred at a lower rate compared to upper and lower limb injuries. Among hand and wrist injuries, fractures were a significant concern, with 45 fractures of hand and wrist bones diagnosed, notably involving the scaphoid bone and other carpal and metacarpal bones. Finger injuries were also frequent, with a total of 144 finger injuries reported, primarily consisting of contusions and fractures, particularly involving the thumb. Shoulder injuries were identified as particularly prevalent, with shoulder instability observed in approximately one-third of cases, along with incidents of superior labrum anterior to posterior (SLAP) tears and supraspinatus tendon tears. Head and neck injuries occurred less frequently, predominantly presenting as lacerations and contusions, with rare instances of brain concussions and spinal cord injuries reported. Overall, 18% of injuries required referral to a hospital for further treatment, with 6.7% of soldiers sustaining moderate injuries and 3.5% requiring surgical intervention. Surgical treatment was often necessitated by shoulder instability, SLAP tears, hand fractures, knee cruciate ligament tears, and other severe injuries. These findings highlight the significant risk of traumatic injuries associated with KM training and emphasize the importance of implementing effective preventive measures and appropriate management protocols to minimize injury incidence and severity among soldiers undergoing combat training [15].

The sport of judo encompasses a spectrum of injuries, with strains, sprains, and contusions being prevalent [16]. These injuries collectively account for a significant percentage of the reported cases, with the knee, shoulder, and elbow collectively representing a substantial percentage of the anatomical locations affected, constituting 17.4%, 15.7%, and 14.2% of injuries, respectively [17]. Interestingly, while there is no significant gender-based difference in shoulder and knee injuries, women tend to experience a higher percentage of elbow injuries compared to men. An in-depth examination of injury types reveals that sprains and soft tissue contusions collectively comprise a considerable proportion of injuries, with sprains occurring in 42.2% of cases and soft tissue contusions in 23.1%. Notably, luxations, predominantly at the shoulder and elbow joints, account for a notable percentage, with 6.8% of injuries attributed to incidents of unconsciousness after strangling and choking techniques. Severity assessments highlight that while a small percentage of injuries necessitate hospital transfer, serious injuries, requiring hospitalization, remain a concern. Shoulder injuries emerge as the most frequent serious injury location, affecting approximately one-third of judokas requiring hospital transfer. Additionally, elbow injuries exhibit a notable proportion of serious cases, comprising 32.3% of injuries necessitating hospitalization. Furthermore, fractures, while less frequent, carry a higher likelihood of severity, with approximately 86.7% of reported cases classified as serious injuries requiring hospitalization. Sprains, despite being the most common injury type, also contribute significantly to the serious injury category, with 44 out of 293 cases classified as severe. Luxations follow a similar trend, with approximately 57.4% of cases classified as serious injuries. In summary, while judo injuries encompass a diverse range of types and locations, preventive measures should particularly focus on addressing the prevalence of shoulder, elbow, and knee injuries, as well as mitigating the severity of luxations and fractures. Additionally, ensuring prompt medical attention for potentially serious injuries, such as neck injuries and concussions, remains imperative to safeguard the well-being of judo practitioners [18].

Brazilian jiu-jitsu (BJJ) has emerged as a popular martial art worldwide, yet there has been a notable absence of medical studies exploring injuries specifically within BJJ competitions. Addressing this gap, a recent study aimed to estimate the incidence of injuries in BJJ competitions and identify the types and mechanisms of injuries associated with competitive BJJ. The findings revealed an injury incidence of 9.2 per 1000 exposures on the day of matches, with 46 injuries recorded out of 5022 exposures, highlighting the prevalence of injuries within this sport [19]. Orthopedic injuries emerged as the most common injury type in an overall total of 47 injuries, constituting a significant majority of 78%. This was followed by costochondral or rib injuries (n=7) and lacerations requiring medical care (n=3). Notably, the elbow was identified as the joint most commonly injured during BJJ competitions, with the arm bar technique being the primary mechanism responsible for these injuries. This specific injury mechanism underscores the importance of recognizing the arm bar as a distinct cause of hyperextension injury to the elbow in sports, particularly in the context of BJJ. Comparative analysis with injury data from judo, taekwondo, wrestling, and mixed martial arts (MMA) revealed that BJJ competitors faced substantially lower risk of injury compared to athletes in these other sports. Despite this relatively lower risk, the prevalence of orthopedic injuries in BJJ competitions underscores the importance of educating participants, referees, and medical personnel about the unique injury mechanisms inherent to BJJ, particularly those affecting the elbow. By raising awareness and understanding of these injury patterns, measures can be implemented to mitigate risks and enhance safety protocols within the sport of BJJ [20].

Gender association

The incidence of neck injuries in sports has been extensively studied, revealing notable differences between male and female athletes. While both genders are susceptible to cervical spine injuries, female athletes often face distinct risk factors and injury patterns compared to their male counterparts [21]. Research indicates that adolescent and female youth karate athletes are at a higher risk of injury than male athletes, suggesting gender-specific vulnerabilities within this sport. Additionally, during the 2012 London Olympic Taekwondo Games, male athletes exhibited a twofold higher risk of injury than females, with concussions exclusively occurring in male participants [6]. This gender disparity in injury rates underscores the importance of understanding and addressing the unique risks faced by female athletes in combat and contact sports. Notably, female athletes have been found to be at a higher risk for cervical strain injuries across various sports compared to males [4]. This elevated risk may be attributed to gender-related differences in muscular composition, highlighting the need for tailored injury prevention strategies that account for physiological variations between men and women. While cervical disc herniation demonstrates equal incidence between genders, it is primarily a degenerative phenomenon unrelated to sports participation, suggesting that other factors contribute to the observed gender differences in neck injuries among athletes. Despite the predominance of male participants in many contact sports, female athletes increasingly engage in these activities, raising questions about their susceptibility to neck injuries. Future studies are warranted to explore whether female athletes face elevated risks of traumatic neck injuries due to congenital narrowing of the spinal canal or heightened ligamentous laxity. Understanding the underlying mechanisms driving these gender-specific injury patterns is crucial for developing targeted interventions and guidelines to promote the safety and well-being of female athletes participating in combat and contact sports. Overall, while the incidence of major traumatic neck injuries in sports predominantly affects male athletes, emerging evidence suggests that female athletes may face unique challenges and vulnerabilities. By identifying and addressing these gender-specific risk factors, stakeholders can work toward creating a safer and more inclusive sporting environment for athletes of all genders.

Clinical presentation

Engaging in sports comes with inherent risks of various injuries, spanning from minor muscle strains to severe conditions like neck fractures, cervical spinal cord injuries, and concussions [22-25]. While individuals with neck fractures may recover fully, restoring neurological function, severe cases can culminate in complete spinal cord injuries, potentially leading to paralysis or even fatalities [26]. Despite strides in protective gear, heightened awareness, and enhanced coaching methods, concerns persist regarding cervical injuries, notably in contact sports like football, soccer, and rugby, as well as in non-contact activities such as gymnastics.

Muscle and bone injuries are common in various combat sports, often resulting from the dynamic and physically demanding nature of these activities [27]. In sports like kickboxing and taekwondo, where kicking and striking techniques are prevalent, often include strains, sprains, and contusions, which can occur during the execution of powerful kicks or blocks. Additionally, the repetitive impact and rotational forces involved in striking techniques can injure limbs and upper extremities. The high prevalence of these injuries underscores the importance of adequate conditioning and proper technique training to minimize the risk of musculoskeletal trauma in combat sports [28]. Furthermore, grappling sports like wrestling, judo, and BJJ involve intricate techniques that require significant physical exertion and force application, predisposing athletes to a different spectrum of muscle and bone injuries. In these disciplines, injuries often occur during throws, takedowns, or submission maneuvers, where athletes may experience sudden impacts or hyperextension of joints. Shoulder and elbow injuries are particularly common due to the repetitive stress placed on these joints during grappling exchanges, such as arm bars or shoulder locks. Additionally, the nature of these sports may also lead to spinal injuries, including strains, sprains, or disc herniations, especially during takedowns or falls.

These injuries manifest in various clinical syndromes, including cervical fractures and dislocations, nerve root or brachial plexus injuries, intervertebral disc lesions, cervical stenosis, acute cervical sprains or strains (like whiplash injury), and transient quadriplegia. Each syndrome presents unique challenges and requires tailored management approaches to ensure optimal outcomes for affected athletes. A neck fracture can occur due to a severe twist or forceful blow to the head or neck region, common in sports involving intense physical contact like football, ice hockey, rugby, and wrestling [29]. Even non-contact activities such as gymnastics pose risks, especially during maneuvers like releasing from the high bar. The cervical spine typically absorbs impact by distributing forces through muscles, intervertebral discs, and bones along its natural curve. However, forceful neck flexion, such as in spear tackling, can strain ligaments or cause bone tears, resulting in various cervical spine injuries, with or without neurological symptoms.

Suspected neck injuries require prompt medical attention to prevent further harm [30]. Moving the affected individual without proper medical assistance should be avoided to maintain cervical spine stability [31]. Recognizing the signs of a neck fracture, including localized pain, stiffness, swelling, bruising, tenderness, and neurological deficits, is crucial. Immediate evaluation and appropriate medical intervention are necessary to minimize complications and optimize recovery [32].

Concussions, a subtype of mild TBI, result from traumatic forces to the head and present immediate symptoms and cognitive impairments [33-35]. Diagnosis relies on subjective evaluation encompassing cognitive and motor assessments, alongside self-reported symptoms such as headache, dizziness, and confusion. Other manifestations span various clinical domains, including somatic, cognitive, emotional, physical, balance impairment, behavioral changes, cognitive impairment, and sleep/wake disturbance. The Centers for Disease Control and Prevention (CDC) estimates that approximately 10% of all sports athletes in the United States suffer from a concussion annually. Among children, collision or contact sports account for 45% of sports- and recreation-related TBIs, with notable incidences in football, soccer, and basketball. Notably, the age group most frequently presenting to emergency departments for sports-related TBIs falls between 10 to 14 years old. Females are observed to have a higher risk of sustaining sports-related TBIs, potentially attributed to factors like lower neck strength and hormonal fluctuations. Athletes with a history of concussion or neck injury have greater odds of presenting with higher symptom scores, indicating a potential need for further investigation into concussion-related domains [36]. Despite increased awareness and preventive measures, SRC remain a significant public health concern, particularly among youth athletes [37]. The prevalence of concussions underscores the importance of ongoing research, education, and injury prevention strategies to mitigate their impact. Efforts aimed at enhancing concussion recognition, management, and rehabilitation protocols are critical to ensuring the safety and well-being of athletes across all levels of participation. Moreover, addressing risk factors contributing to higher susceptibility among certain demographics, such as females and specific age groups, is essential for implementing targeted interventions and promoting safer sports environments. By fostering a multidisciplinary approach involving healthcare professionals, coaches, parents, and athletes themselves, comprehensive strategies can be developed to minimize the incidence and severity of SRC while optimizing the long-term health outcomes of those affected.

Diagnostic approach

Traumatic injuries to the spine present a significant challenge due to their potential for long-term complications. Rapid diagnosis and effective management of spinal instability are crucial for preventing further neurological damage and optimizing patient outcomes. In the trauma setting, determining the necessity of spinal imaging is pivotal for guiding treatment strategies. Various imaging techniques play distinct roles in evaluating spinal injuries.

Initially, plain radiographs serve as a screening tool, detecting bony abnormalities. However, with advancements in technology, multidetector computed tomography (CT) has become the preferred method for detailed assessment of bony structures, particularly useful for evaluating fractures and spinal alignment. Conversely, magnetic resonance imaging (MRI) offers superior soft tissue contrast, essential for assessing spinal cord injuries, ligament damage, and other soft tissue abnormalities [38]. Upper cervical injuries, often stemming from high-energy trauma like sudden spinal movements or forceful blows to the head, encompass a spectrum of injuries affecting the skull base, atlas, and axis. While CT is increasingly favored for high-energy injuries, MRI can complement it by providing indirect evidence of bony trauma, such as bone marrow edema detected in T2-weighted images, thus enhancing diagnostic accuracy and informing treatment decisions [39,40]. Despite the widespread use of CT, false-positive results can occur due to various factors, including motion artifacts, anatomical variations, or technical errors. These inaccuracies can potentially lead to mismanagement, with patients undergoing unnecessary treatments. For example, motion artifacts or vascular variants observed on CT images may mimic cervical spine fractures, resulting in unwarranted interventions. Clinical re-evaluation becomes crucial when there is a discrepancy between clinical findings and radiological imaging to avoid unnecessary procedures and patient transfers.

MRI offers advantages in assessing the soft tissue component of spinal trauma, helping to identify active bone lesions and preventing unnecessary interventions [41]. BLADE sequences in MRI have shown promise in reducing motion, truncation, and flow artifacts, thereby improving image quality and diagnostic accuracy. Sagittal cervical spine imaging with BLADE sequences enhances the contrast between the spinal cord and cerebrospinal fluid (CSF), reduces CSF-flow artifacts, and improves the visualization of surrounding structures. While T2-BLADE in axial orientation may lead to blurring of the subarachnoid space due to CSF flow effects, it still provides valuable information for spinal cord interpretation, albeit with some limitations compared to traditional Turbo Spin Echo (TSE) sequences.

Treatment strategies

In sports medicine, managing injuries effectively begins with prompt and appropriate emergency care, particularly focusing on stabilization to prevent further harm. For instance, in cases of neck injuries, immediate attention is given to immobilizing the spine to minimize the risk of exacerbating spinal cord damage [42]. This often involves techniques such as cervical collar placement and backboard immobilization, followed by careful transportation to a medical facility for further evaluation and treatment [43]. By prioritizing stabilization in the acute phase, healthcare providers can mitigate the potential for catastrophic outcomes and lay the groundwork for subsequent interventions [30].

Once the patient's condition is stabilized, attention turns to managing superficial injuries such as lacerations and abrasions. A systematic approach is employed, starting with assessing the patient's airway, breathing, and circulation (ABCs) to address any life-threatening concerns. Hemostasis is crucial to control bleeding, typically achieved through direct pressure and, if necessary, the use of vasoconstrictive agents. Following hemostasis, thorough wound inspection and exploration are performed to identify any associated injuries, with local anesthesia administered for pain management. Wound irrigation, using solutions like normal saline, helps minimize infection risk before selecting an appropriate closure technique, ranging from sutures to adhesive glues, depending on wound characteristics [44].

In contrast, critical injuries such as severe cervical spine trauma demand a more nuanced approach, balancing the need for timely intervention with the risks associated with surgical management. Individualized assessments, including reliable neurological examinations and imaging studies, guide decisions regarding spine clearance and the timing of surgical intervention. Early surgery within 24 hours may be warranted to achieve definitive spinal cord decompression and stabilization, with a focus on optimizing oxygenation and perfusion while mitigating systemic complications. However, the use of certain interventions, such as high-dose methylprednisolone, is now discouraged due to associated adverse effects [45].

Surgical management plays a pivotal role in addressing complex sports injuries, particularly those involving severe cervical spine trauma or significant musculoskeletal damage. Surgical techniques aim to restore anatomical integrity and function while minimizing long-term sequelae. For instance, anterior cervical discectomy and fusion (ACDF) may be employed to address cervical spine injuries in athletes, with meticulous attention to fusion integrity and postoperative rehabilitation protocols. Despite the potential for successful outcomes, surgical interventions carry inherent risks, including infection, bleeding, and prolonged recovery periods, necessitating careful consideration of patient-specific factors and informed decision-making [46].

Recovery and rehabilitation

The recovery journey following sports-related injuries includes a spectrum of challenges and timelines, influenced by various factors unique to each athlete's circumstances. Whether it is the aftermath of a concussion, the rehabilitation period post-cervical spine surgery, or the recovery process following muscle strains or limb fractures, understanding the nuances of each phase is crucial for optimizing outcomes and facilitating a safe return to sports participation.

Beginning with the management of sport-related concussions (SRC), the focus lies on the intricate balance between symptom resolution, cognitive recovery, and the eventual return to school or sports activities (RTL and RTS, respectively). Studies reveal rapid cognitive recovery, with most achieving full RTL in 8.3 days. However, RTS takes longer, averaging about 19.8 days. This difference reflects SRC recovery's multifaceted nature. Factors like symptom severity, post-injury activity, and access to medical care influence the process. Moreover, previous concussions can prolong recovery [34]. Studies show that females or younger athletes may face longer recovery periods. Each athlete's situation is unique. Thus, tailored assessment and management are crucial. By navigating these complexities, optimal outcomes can be achieved, facilitating a safe return to academic and athletic activities for those recovering from concussive injuries [47].

Moving forward, the recovery period following cervical spine surgery in athletes returning to martial arts presents a unique set of challenges and considerations. While a significant proportion of athletes may resume activity within the first six months post-operation, there exists a subgroup requiring extended rehabilitation due to the complexity or severity of their injuries. This underscores the importance of comprehensive preoperative assessments, meticulous surgical planning, and personalized rehabilitation protocols tailored to the specific needs and injury profiles of athletes participating in high-contact sports like martial arts [48].

In the realm of cervical spine injuries, particularly those necessitating ACDF, the decision of when athletes can safely return to contact sports remains a contentious issue. However, studies focusing on professional athletes undergoing single-level ACDF demonstrate promising outcomes, with the majority successfully resuming their sports within a relatively short timeframe postoperatively. Nevertheless, long-term considerations such as the risk of adjacent segment disease and persistent cord hyperintensity necessitate continued monitoring and research to refine guidelines and ensure the safety of athletes returning to high-contact sports activities [49].

Finally, the recovery period for muscle injuries and limb fractures following sports-related incidents underscores the importance of a comprehensive approach encompassing rest, physiotherapy, and gradual return to activity. While minor injuries may heal relatively quickly with conservative management, more severe or complex cases may require surgical intervention and a more prolonged rehabilitation period. By addressing both the acute and rehabilitative phases of injury management, healthcare professionals can optimize outcomes and facilitate a safe return to sports participation for athletes across various disciplines.

Quality of life

Severe sports injuries can drastically alter an athlete's quality of life, affecting their physical health and psychological well-being in profound ways. Conditions such as tetraplegia, CTE, and chronic neck pain and muscle injuries present unique challenges that can significantly diminish overall well-being and disrupt daily life [50].

Living with CTE can profoundly affect an athlete's quality of life, with symptoms progressively worsening over time. Initially, individuals may experience subtle cognitive impairments, mood swings, and behavioral changes that disrupt their ability to function effectively in their personal and professional lives. As the disease advances, these symptoms often intensify, leading to significant functional limitations and a diminishing capacity to participate in activities that once brought them joy and fulfillment. Memory loss, executive dysfunction, and emotional instability can strain relationships, impair decision-making abilities, and make daily tasks increasingly challenging to manage independently [51].

Chronic neck pain and muscle injuries can also greatly affect the quality of life for athletes. Constant discomfort and limited mobility resulting from these conditions can hinder participation in sports and other physical activities, leading to a loss of enjoyment and fulfillment. Individuals may experience persistent pain, stiffness, and fatigue, impacting their ability to perform daily tasks and engage in social or recreational pursuits. Additionally, the ongoing nature of these injuries can contribute to feelings of frustration and hopelessness, as well as challenges in maintaining work or academic responsibilities. Despite attempts to manage symptoms through various treatments and therapies, chronic neck pain and muscle injuries can have a significant detrimental effect on an athlete's overall quality of life, affecting both physical and emotional well-being.

The psychological toll of severe sports injuries is significant and often overlooked. Athletes facing tetraplegia, CTE or chronic neck pain, and muscle injuries may experience depression, anxiety, and feelings of grief over the loss of their previous abilities and lifestyle. Coping with these emotional challenges alongside physical limitations can be overwhelming, straining relationships and diminishing overall well-being. Despite efforts to provide support and resources, athletes grappling with severe sports injuries face a daunting journey marked by profound challenges to their physical, emotional, and social health.

Prevention

Injuries resulting from sports activities, particularly combat sports, pose significant concerns for athletes, coaches, and medical professionals alike. Combat sports, such as boxing, karate, and taekwondo, involve physical contact and intense physical exertion, increasing the risk of various injuries, including head and neck injuries. Addressing these risks and implementing effective strategies to reduce the incidence and severity of injuries is paramount for ensuring the safety and well-being of athletes.

Several studies and initiatives have focused on understanding and mitigating the risks associated with combat sports injuries. For instance, the implementation of new competition categories in U21 karate has been associated with a significant reduction in injury rates, highlighting the potential efficacy of modifying competition formats to enhance safety [5]. Additionally, advancements in equipment design, such as the development of novel Cervical Spine Protection Devices (CSPDs) for football players, demonstrate efforts to minimize the risk of head and neck injuries by optimizing protective gear [52].

Concussion prevention strategies have also been a focal point in combat sports safety measures. Recommendations for concussion education programs in both pediatric and adult amateur athletes underscore the importance of raising awareness and promoting informed decision-making to mitigate the risk of head injuries [53]. Moreover, the endorsement of head protective equipment, particularly newer football helmet technology, reflects the substantial evidence supporting the effectiveness of such measures in reducing the incidence of concussions and severe head injuries [54].

Rule modifications play a crucial role in mitigating the risk of injuries in sports, particularly combat sports where physical contact is inherent. Over the years, governing bodies and sports organizations have implemented various rule changes aimed at enhancing player safety and reducing the incidence of injuries [55]. These modifications often target specific aspects of gameplay, such as tackling techniques, equipment usage, and permissible maneuvers, with the ultimate goal of minimizing the risk of harm to athletes. In combat sports like boxing and MMA, rule modifications frequently focus on regulating the intensity and frequency of strikes, particularly those targeting the head and neck regions. For example, weight classes and time limits are established to ensure that bouts are conducted in a manner that minimizes the risk of excessive force and prolonged exposure to potential injury. Additionally, referees and officials are trained to enforce rules that prohibit certain dangerous maneuvers, such as strikes to the back of the head or spine, which could result in catastrophic injuries [56].

In team sports like rugby and American football, rule modifications often address aspects of gameplay that are associated with high-impact collisions and traumatic injuries. For instance, in rugby, recent rule changes have aimed to reduce the risk of head and neck injuries during scrums by implementing stricter engagement protocols and emphasizing proper technique among players. Similarly, in American football, the adoption of rules like the "Crown-of-the-Helmet Rule" aims to discourage players from using dangerous tackling techniques that increase the risk of head and neck injuries [57].

Moreover, rule modifications are not limited to addressing in-game actions but also extend to equipment standards and player conduct. Sports organizations frequently update equipment regulations to ensure that athletes are provided with adequate protection against potential injuries. Additionally, codes of conduct and disciplinary measures are enforced to discourage unsafe behavior on and off the field, promoting a culture of respect and sportsmanship among competitors [6,58].

## Conclusions

In conclusion, the gap in understanding between injuries in regular sports and combat sports is significant, particularly regarding cervical spine injuries. While there is extensive knowledge on injury diagnosis and treatment in regular sports, combat sports lack comprehensive resources, making the frequency and severity of cervical spine injuries especially concerning. This lack of data hinders the development of targeted preventive measures, as combat sports involve unique risks and dynamics that differ from other sports. Without a clear understanding of injury patterns and long-term effects in combat sports, effective risk-reduction strategies remain elusive. Therefore, prioritizing research on cervical spine injuries in combat sports is essential to ensure athlete safety and well-being.
